# Migration and Forms of Metals in Bottom Sediments of Czerniakowskie Lake

**DOI:** 10.1007/s00128-012-0881-7

**Published:** 2012-12-02

**Authors:** Małgorzata Wojtkowska

**Affiliations:** Faculty of Environmental Engineering, Warsaw University of Technology, ul. Nowowiejska 20, 00-653 Warsaw, Poland

**Keywords:** Metals, Sediments, Forms, Sequential extraction procedure

## Abstract

The goal of the research was to determine the concentration of Zn, Cu, Pb and Cd forms in the bottom sediments of Czerniakowskie Lake situated within the boundaries of Warsaw. Sediment cores collected for examination purposes were divided into sections, which were approximately 5 cm thick and were analysed to determine the content of metal forms. In the investigated sediments metals were characterized by different percentage in the individual fractions. Speciation analysis indicated that the following fractions were dominant in the bottom sediments of Czerniakowskie Lake: the fraction connected with iron and manganese oxides (for lead and zinc), the fraction connected with organic matter (for copper), the carbonate fraction and the fraction connected with iron and manganese oxides (for cadmium).

Metals migrate between individual components of the environment and change their chemical forms and bioavailability. Their presence should be considered as a risk for the existence of ecosystems, irrespectively of the form of their occurrence. There are many factors in the environment, which trigger the transformation of metals into ionic forms, which are the most dangerous form for living organisms. Metals discharge into water precipitate and accumulate in bottom sediments, from where they can be released as a result of changes in physical/chemical and biological conditions in the bottom layer of water (Weng and chen 2000). Transport of metals in bottom sediments depends on many factors resulting from the nature of both sediments and metals. The following properties of metals have an influence on their transport in the sediments: ionic charge, ionic radius, ion complexing power, oxidation state, hydration state. The bonding power of metals in the bottom sediments decreases with the increase in the pH value of the environment. Ions with higher charge (Al^3+^) have stronger bonds than less charged ions (Cu^2+^). In case of different ions with the same charge state the key factors determining the accumulation of metals in the sediments may include the ionic radius and hydration state. Ions with greater radius produce smaller electric field and are less hydrated and consequently migrate easier (Dube et al. 2001; Mackenzie 2005). Exchangeable sorption is the most important type of metal bond. This type of metal bonding in sediments is a reversible process and its speed depends on pH, the type and content of organic matter and colloids, the type and concentration of anions present in the environment and the ambient temperature (Dube et al. 2001). In the process of chemical sorption metal ions are deactivated as a result of their precipitation or co-precipitation into insoluble forms. These processes depend on pH value and carbonate or chloride concentration. Metals deposited in the bottom sediments are not bonded permanently and therefore can be released back into the water environment if conditions change (e.g. pH or redox potential) or in the presence of organic chelates (Dube et al. 2001).

Determination of total metal concentration helps demonstrate the degree of the environmental degradation. However, it does not indicate the level of metal bioavailability. Speciation analysis enables the identification of chemical forms of metals in the sediment. This type of analysis provides the possibility to identify and determine the chemical forms of a given metal in a sample. For analytical purposes, Tessier distinguished five fractions, in which metals are deposited in ion exchangeable, carbonate, adsorptive, organic and residual forms (Wiechuła 2004; Rao et al. 2008). The determination of metal content in the bottom sediments can be used for an ecotoxicological assessment of water reservoirs. The metal content in sediments resulting from long-term sedimentation processes makes it possible to obtain an integrated picture of the reservoir contamination. It is not possible to obtain such a picture from water analysis because the metal content levels in water can vary substantially as a result of for example increased inflow of wastewater (Bojakowska 1994). In the process of assessing metal concentrations and their chemical forms it is essential to take into consideration the existing conditions as well as physical and chemical properties of the sediment, which have an impact on the mobility of trace elements and their bioavailability. In this study, the total concentrations and the concentrations of different forms in the sediments were determined with respect to the background levels (Table 1). The impact of various pollution sources located within a direct catchment area of the lake was also taken into account (Bojakowska 1994). Since 1987 the site has been a water and landscape reserve with the area of around 45 hectares including ca. 20 hectares of the lake area. The original structure of watercourses on the inundation terrace has become obliterated due to the residential development along Czerniakowska street and Bernardyńska street, channelling of local inflows into collectors, construction of a heat pipeline from Siekierki Heat and Power Station, and other anthropogenic activities which, although taking place away from the water reservoir discussed in this paper, have an indirect impact on the lake by changing the ground water circulation routes. Complete vanishing of flows in Czerniakowskie Lake bed resulted in its transformation into a gradually disappearing reservoir with no drainage or outflow route (Wojtkowska 2002).

## Materials and Methods

**Fig. 1:**
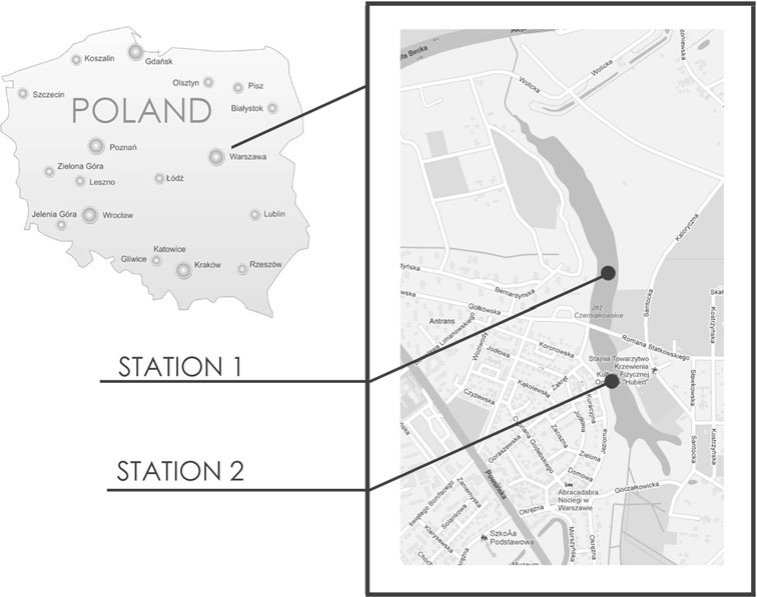
Location of the studied stations in the Czerniakowskie Lake

The research focused on the sediments from Czerniakowskie Lake (Fig. 1). There were three runs of the research. Two bottom sediment cores were collected (one from the northern part of the lake, i.e. station 1, and the other from the southern part, i.e. station 2). The cores were divided into 5 cm thick layers. Five and six layers were distinguished in the sediment profiles collected at station 1 and 2, respectively. The following properties were determined in the sediment samples: pH, conductivity, chlorides, alkalinity and organic substances. The following metals were determined in each layer: Zn, Cu, Pb and Cd. Total metal concentrations in sediment samples were determined by means of total extraction with HNO_3_ and HClO_4_ (1:3) in a Teflon bomb. A modified sequential Tessier scheme (Wiechuła 2004; Rao et al. 2008) was employed to perform the speciation of metals in the sediments. All cations were analyzed using flame atomic absorption spectroscopy (Philips). Trace quantities of metals were determined by means of graphite furnace atomic absorption spectroscopy. Metal concentrations in the supernatant liquid were measured with a flame atomic absorption spectrometry (FAAS). The same procedure without samples was used as a control. Three measurements were conducted for each sample. Quality assurance and quality control (QA/QC) for metals in sediment samples were estimated by determining metal concentrations in the Merck Standard solutions (Merck, Darmstadt, Germany). The detection limit was calculated based on the estimated instrumental detection limit assuming that 1 g of a sample is digested or diluted to 100 mL. Detection limits (mg/kg of dry matter) for Cu, Pb and Zn were: 0.001; 0.003 and 0.001, respectively (Table 1).

**Table 1:** Metal content in the environment—background levels

Metal	Soil and sediments (mg/kg)
Zinc	30–125
Cadmium	0.2–1.0
Copper	10–35
Lead	25–40

## Results and Discussion

The bottom sediment cores collected from Czerniakowskie Lake show quite distinct composition of chemical indicators, as demonstrated in Table 2. The values of individual indicators determined for the two sediment cores differed significantly. However, the observed trends in the changes were rather similar at both stations (Table 2). The pH range detected for the individual layers did not change substantially: it varied from 7.67 to 8.60 for samples collected at station 1, whereas at station 2 the pH range was slightly narrower, namely 7.98–8.4. The lowest pH value was recorded in the surface layer of the sediment at both stations. The alkalinity of the sediments varied, showing higher values in the surface layers. Interestingly, the values decreased in the second layer and increased again with depth. The alkalinity ranged from 7.7 to 10 mmol/dm^3^ at station 1 and from 9.1 to 12.5 mmol/dm^3^ at station 2. The content of chlorides and organic matter as well as hydration in individual layers of sediments decreased with depth (Fig. 1).

**Table 2:** Physical and chemical characteristics of sediments in Czerniakowskie Lake

Station	Depth (cm)	pH	Spec. conduct. (μS/cm)	Alkalinity (mmol/dm^3^)	Chlorides (mg/dm^3^)	Org. matter (%)	Hydration (%)
1	0–5	7.67	1,658	7.7	240	14.05	41.6
	5–10	8.40	1,870	6.2	160	12.49	24.6
	10–15	8.60	1,791	6.7	120	11.89	30.2
	15–20	7.90	1,652	10.0	50	10.11	24.4
	20–25	8.45	1,154	9.0	62.5	8.85	18.6
2	0–5	7.98	2,070	12.5	330	22.29	21.5
	5–10	8.20	2,200	9.1	320	20.44	23.3
	10–15	8.41	6,370	10.4	280	18.58	17.0
	15–20	8.38	7,620	11.5	240	17.73	21.5
	20–25	8.3	1,390	10.0	180	15.19	8.4
	25–30	8.20	3,490	10.0	160	13.99	22.7

**Fig. 2:**
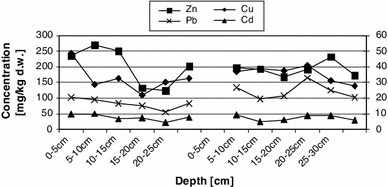
Total concentration of metals in sediment of Czerniakowskie Lake

Total concentrations of zinc, copper, lead and cadmium in the sediment cores decreased with depth (Table 3). According to the data presented in the previous study (Bojakowska et al. 2006), the natural content of zinc in sediments ranges from 30 to 125 mg/kg. The concentrations observed in the bottom sediments of Czerniakowskie Lake significantly exceeded the expected levels of natural background. Total concentrations of copper, lead and cadmium showed the same pattern: they exceeded the natural values of the sediment environment by 10–35, 25–40 and 0.2–1.0 mg/kg, respectively. The results obtained in this study allowed for the classification of the analysed sediments among sediments contaminated with zinc, copper, lead and cadmium. However, basing on this study it was impossible to identify a uniform changeability pattern for contents of analysed metals at both stations (Fig. 2). Clear differences were found between metal concentrations in the two cores of sediments. Higher concentrations of Zn, Cu and Cd were recorded at station 1, in particular in the surface layer, whereas the detected concentration of lead was higher at station 2. The highest zinc levels were found in layers: 5–10 and 10–15 cm at station 1, and in the first two layers at station 2. In case of copper, a significant correlation was found between the Cu content and the content of organic matter in the individual layers. The highest Cu levels were found in layer 0–5 cm at station 1 and layer 15–20 cm at station 2; in the latter case the organic matter content was higher as well. Differences in copper levels found in individual sediment layers were insignificant. Cadmium showed the largest variations of its content in individual layers. However, the concentration of this metal in the layers of the samples collected at both stations was similar. Lead content showed a positive correlation with the chloride content. In general, it may be concluded that organic matter, hydration, chlorides and metal concentrations reached their lowest values in the deepest layer of the sediments. A similar pattern was observed in another research conducted in southern Poland (Dube et al. 2001).

**Table 3:** Total concentration of metals (mg/kg dry matter)—medium, minimum and maximum values

	Zinc		Copper		Lead		Cadmium	
	1	2	1	2	1	2	1	2
Medium	202.0	197.5	32.4	36.4	82.4	129.3	7.6	7.7
Max	269.1	232.3	48.5	40.9	102.2	164.3	10.0	9.4
Min	123.7	167.4	21.8	31.0	55.4	98.3	4.3	4.9
Median	234.8	192.9	30.1	37.4	83.0	125.3	7.4	8.6

**Fig. 3:**
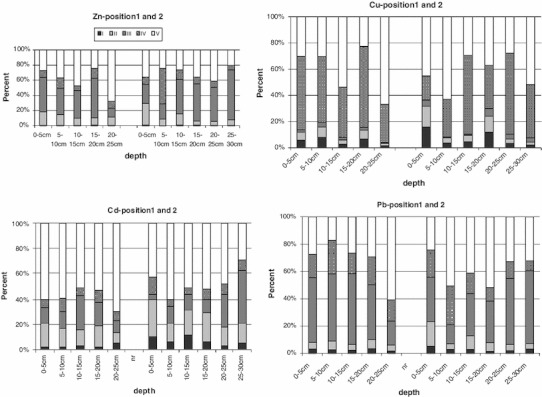
Percentage of Cu, Zn, Cd and Pb forms in the individual layers of sediments

The research revealed different tendencies in bonding of Zn, Cu, Pb and Cd by the bottom sediments of the lake (Fig. 3). The profile of metal distribution among five fractions is depicted in the diagrams. The speciation analysis demonstrated that the fraction of hydrated iron and manganese oxides was a dominant form of zinc occurrence (40 %). Lead showed a similar pattern (39 %), bonding mainly with Fe–Mn oxides. Copper was proved to have the highest affinity for the organic fraction (45 %). Only cadmium did not exhibit a clear capability to stay within one fraction. The highest percentage of this element was recorded in the residual fraction. In the group of the bioavailable fractions, cadmium was strongly bonded with carbonates (17 %) and Fe–Mn oxides (17 %). The results produced by speciation analysis prove the bonding of metals in the sediments of the lake in the forms characterised by substantial mobility and bioavailability. A sequential analysis indicated that the retained trace metals were in mobile forms and could be released if ambient conditions changed. Similar results were obtained by Marsalek and Marsalek (1997). The natural content of the metals in the bottom sediments of a water reservoir is quite high and it depends on how the direct catchment area is developed. Increased metal content indicates the anthropogenic contamination of Czerniakowskie Lake environment. The bioavailability of metals depends, to a greater extent, on the form in which a given metal occurs rather than on the total metal concentration in the bottom sediment. Metals occurring in mobile forms show highest toxicity. Easy-to-decompose compounds, which quickly release the metals bonded in them into the environment, are another form of metal occurrence, which is hazardous to ecosystems. The presence of the mobile form of cadmium indicates the possible release of Cd into the water environment, especially in situations, when pH level decreases. The parallel occurrence of copper and organic matter suggests the possibility of remobilisation of this metal if the organic matter deposited in the bottom sediments decomposes. Additionally, the presence of lead and zinc mainly in the adsorption fraction suggests the possibility of Pb migration into the bottom layer of the lake water in the changing oxidation/reduction conditions at the water/sediment interface (Weng and Chen 2000; Alvares et al. 2001; Bordas and Bourg 2001; Lindström 2001). The particularly large concentrations of metals in the surface layer of the bottom sediments indicate a constant inflow of these pollutants to Czerniakowskie Lake. Both precipitation and surface run-off from adjacent roadways may be a source of the metals in the lake, since the reservoir is located in a high traffic area. The chemical profile of the bottom sediments of Czerniakowskie Lake shows substantial relationship between macroelements and metals. The variations in copper content were connected with the amount of organic matter, whereas the variations in zinc and lead contents were related to the carbonate and chloride content, respectively. Mobility and availability of the analysed metals for the biomass of the lake will depend on their co-occurrence. Copper, which is considered as a metal of low mobility, can displace other metals bonded in individual fractions, whereas metals with low affinity for solid phase (Zn, Cd, Ni) will be displaced by cations with high affinity (Cu, Cr), increasing their bioavailability (Paszko 2006).

The results of the research not only prove the need for monitoring the sediments of Czerniakowskie Lake in terms of total concentrations, but also indicate that it is necessary to determine the forms of metal bonding in the sediments.
